# Laboratory Study of Collisionless Magnetic Reconnection

**DOI:** 10.1007/s11214-023-01024-3

**Published:** 2023-11-15

**Authors:** H. Ji, J. Yoo, W. Fox, M. Yamada, M. Argall, J. Egedal, Y.-H. Liu, R. Wilder, S. Eriksson, W. Daughton, K. Bergstedt, S. Bose, J. Burch, R. Torbert, J. Ng, L.-J. Chen

**Affiliations:** 1 Department of Astrophysical Sciences, Princeton University, 4 Ivy Lane, Princeton, 08544 New Jersey USA; 2 Princeton Plasma Physics Laboratory, P.O. Box 451, Princeton, 08543 New Jersey USA; 3 Institute for the Study of Earth, Oceans, and Space, University of New Hampshire, 8 College Road, Durham, 03824 New Hampshire USA; 4 Department of Physics, University of Wisconsin - Madison, 1150 University Avenue, Madison, 53706 Wisconsin USA; 5 Department of Physics and Astronomy, Dartmouth College, 17 Fayerweather Hill Road, Hanover, 03755 New Hampshire USA; 6 Department of Physics, University of Texas at Arlington, 701 S. Nedderman Drive, Arlington, 76019 Texas USA; 7 Laboratory for Atmospheric and Space Physics, University of Colorado at Boulder, 1234 Innovation Drive, Boulder, 80303 Colorado USA; 8 Los Alamos National Laboratory, P.O. Box 1663, Los Alamos, 87545 New Mexico USA; 9 Southwest Research Institute, 6220 Culebra Road, San Antonio, 78238 Texas USA; 10 Department of Astronomy, University of Maryland, 4296 Stadium Drive, College Park, 20742 Maryland USA; 11 Goddard Space Flight Center, Mail Code 130, Greenbelt, 20771 Maryland USA

**Keywords:** Magnetic reconnection, Laboratory experiment, Magnetospheric MultiScale

## Abstract

A concise review is given on the past two decades’ results from laboratory experiments on collisionless magnetic reconnection in direct relation with space measurements, especially by the Magnetospheric Multiscale (MMS) mission. Highlights include spatial structures of electromagnetic fields in ion and electron diffusion regions as a function of upstream symmetry and guide field strength, energy conversion and partitioning from magnetic field to ions and electrons including particle acceleration, electrostatic and electromagnetic kinetic plasma waves with various wavelengths, and plasmoid-mediated multiscale reconnection. Combined with the progress in theoretical, numerical, and observational studies, the physics foundation of fast reconnection in collisionless plasmas has been largely established, at least within the parameter ranges and spatial scales that were studied. Immediate and long-term future opportunities based on multiscale experiments and space missions supported by exascale computation are discussed, including dissipation by kinetic plasma waves, particle heating and acceleration, and multiscale physics across fluid and kinetic scales.

## Introduction

The history of laboratory studies of magnetic reconnection goes back to 1960s (e.g. Bratenahl and Yeates 1970), not long after the development of early theoretical models (Sweet 1958; Parker 1957; Dungey 1961; Petschek 1964). As briefly reviewed by Yamada et al. (2010), these early experiments were motivated by solar flares, and were carried out in a collision-dominated MHD regime at low Lundquist numbers ($S<10$). The subsequent landmark experiments performed by Stenzel and Gekelman (1979) were also at low Lundquist numbers ($S<10$), but in the electron-only reconnection regime where ions are unmagnetized even with a strong guide field. While these experiments provided insights into the rich physics of magnetic reconnection in relatively collisional regimes, they are not directly relevant to collisionless reconnection in space, which is the focus of this book; therefore, they are not included in this short review paper except in a few relevant places.

Modern reconnection experiments started with merging magnetized plasmas (Yamada et al. 1990; Ono et al. 1993; Brown 1999) using technologies developed during nuclear fusion research. These were followed by driven reconnection experiments in an axisymmetric geometry: Magnetic Reconnection Experiment or MRX (Yamada et al. 1997), Versatile Toroidal Facility or VTF (Egedal et al. 2000), and Terrestrial Reconnection Experiment or TREX (Olson et al. 2016); and in a linear geometry: Rotating Wall Experiment (RWX) (Bergerson et al. 2006), Reconnection Scaling Experiment (RSX) (Intrator et al. 2009), and the more recent Phase Space Mapping experiment (PHASMA) (Shi et al. 2022). There exist also experiments using Z-pinches (Hare et al. 2017) and lasers (e.g. Chien et al. 2023) in relevant conditions. A non-exhaustive list of relevant experiments to this paper are listed in Table 1, including the upcoming Facility for Laboratory Reconnection Experiments or FLARE (Ji et al. 2018, 2022). Many of these experiments were able to reach higher Lundquist number, up to $S\sim 10^{3}$, with magnetized ions. As a result, plasma conditions local to the reconnecting current sheets in these experiments were nearly collisionless, motivating quantitative comparisons with *in-situ* measurements by spacecraft in near-Earth space as well as predictions by Particle-In-Cell (PIC) kinetic simulations.

**Table 1:** A non-exhaustive list of relevant experiments on collisionless reconnection

Facility / location	Main features / topics	Main or relevant references
Linear device / UCLA	electron-only / waves, non-thermal electrons, plasmoids	Stenzel and Gekelman (1979), Gekelman and Stenzel (1984, 1985), Stenzel et al. (1986)
TS-3/4 / U. Tokyo	toroidal plasma merging / heating, plasmoids	Yamada et al. (1990), Ono et al. (1993, 2011)
MRX / Princeton	axisymmetric current sheet, toroidal plasma merging / reconnection rate, structure, heating, waves, 3D, plasmoids	Yamada et al. (1997, 2006, 2014, 2018), Ji et al. (1998, 2004, 2005, 2008), Hsu et al. (2000), Carter et al. (2001), Ren et al. (2005, 2008), Kulsrud et al. (2005), Tharp et al. (2012), Lawrence et al. (2013), Dorfman et al. (2013, 2014), Yoo et al. (2013, 2014b, 2018, 2023), Jara-Almonte et al. (2016), Fox et al. (2017, 2018), Bose et al. (2023)
SSX / Swarthmore	toroidal plasma merging / heating	Brown (1999), Brown et al. (2002, 2006)
VTF / MIT	axisymmetric current sheet, strong guide field / structure, heating, waves, onset	Egedal et al. (2000, 2003), Egedal and Fasoli (2001), Stark et al. (2005), Katz et al. (2010), Fox et al. (2008, 2010, 2012)
RSX / Los Alamos National Lab	linear plasma merging / onset, 3D	Intrator et al. (2009)
RWX / U. Wisconsin	liner geometry / onset	Bergerson et al. (2006)
TREX / U. Wisconsin	axisymmetric current sheet / structure, plasmoids	Olson et al. (2016, 2021), Greess et al. (2021)
MAGPIE / Imperial	Z-pinch / heating, plasmoids	Hare et al. (2017)
PHASMA / West Virginia U.	linear plasma merging, electron-only / heating	Shi et al. (2022)
Capacitor coil powered by laser / U. Rochester	current sheet / electron acceleration, waves	Chien et al. (2023), Zhang et al. (2023)
FLARE / Princeton	axisymmetric current sheet / multiscale	Ji et al. (2018, 2022)

The topics on magnetic reconnection for such comparative research include kinetic structures of diffusion regions, energy conversion from magnetic field to plasma, various plasma wave activities, and multiscale reconnection via plasmoid instability of reconnecting current sheets. This paper concisely reviews results from these comparative research activities and highlights several recent achievements, especially in relation to the Magnetospheric Multiscale (MMS) mission. Summary of magnetic reconnection research in a broader scope can be found in review papers by Zweibel and Yamada (2009) and Yamada et al. (2010), as well as in more recent reviews (Yamada 2022; Ji et al. 2022). The latter review paper especially focuses on the future development of magnetic reconnection research by emphasizing its multiscale nature.

The rest of this review is organized into the following sections: kinetic structures of reconnecting diffusion regions in Sect. 2 including both the ion and electron diffusion regions (IDR and EDR), reconnection energetics in Sect. 3, plasma waves in Sect. 4, plasmoids during reconnection in Sect. 5, and the future outlook in Sect. 6.

## Kinetic Structures of Diffusion Regions

It is interesting that detailed studies of magnetic reconnection based on *in-situ* measurements in modern experiments (e.g. Yamada et al. 1997) and in space (e.g. Fujimoto et al. 1996; Øieroset et al. 1997) began nearly contemporaneously with detecting kinetic structures of diffusion regions near the X-line, as the research focus was the origin of fast reconnection in collisionless plasmas. The origin of kinetic structures which support the reconnection electric field in collisionless plasmas can be understood via the generalized Ohm’s law,

1 $$ \boldsymbol{E} + \boldsymbol{V} \times \boldsymbol{B} = \eta _{s} \boldsymbol{j} + \frac{\boldsymbol{j} \times \boldsymbol{B}}{en} - \frac{\boldsymbol{\nabla}p_{e}}{en}- \frac{\boldsymbol{\nabla}\cdot \boldsymbol{\Pi}_{e}}{en}- \frac{m_{e}}{e} \frac{d \boldsymbol{V}_{e}}{dt}, $$

where $\boldsymbol{E}$, $\boldsymbol{V}$, $\boldsymbol{B}$, and $\boldsymbol{j}$ are electric field, velocity, magnetic field, and current density, respectively, and $\eta _{s}$ is the Spitzer resistivity. $n$, $\boldsymbol{V}_{e}$, $m_{e}$, and $e$ are the electron density, fluid velocity, mass, and charge, respectively. The full electron pressure tensor is expressed as a sum of a diagonal isotropic pressure tensor and a stress tensor which includes an off-diagonal pressure tensor: $\boldsymbol{P}_{e} \equiv p_{e} \boldsymbol{I} + \boldsymbol{\Pi}_{e}$ where $\boldsymbol{I}$ is the unit tensor. The RHS of Eq. (1) represents non-ideal-MHD electric field in diffusion regions where $\boldsymbol{V} \times \boldsymbol{B}$ diminishes while $\boldsymbol{E}$ remains large for fast reconnection. Each of these non-ideal-MHD terms is associated with a spatial structure in steady state on the corresponding scale in electromagnetic field or electron quantities.

**Fig. 1:**
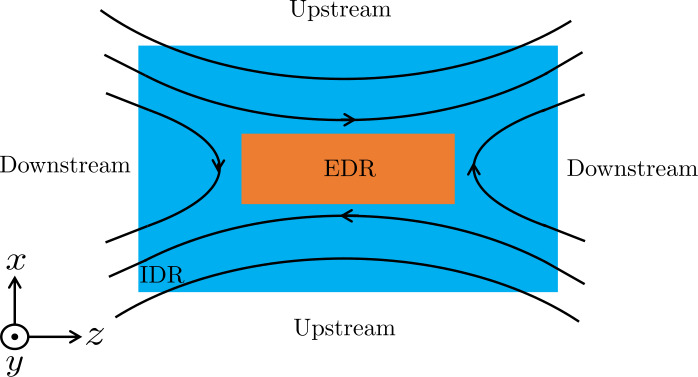
Schematics of magnetic reconnection geometry and coordinates. Plasma flows in from upstream with oppositely directed magnetic field components towards the ion diffusion region (IDR), where ions become demagnetized, before reaching the electron diffusion region (EDR), where electrons become demagnetized and reconnection occurs. The reconnected plasma flows downstream. Unless stated explicitly, the reconnection plane $(z,x)$ is defined so that $z$ is along the reconnecting field component and $x$ is the direction across the current sheet. $y$ completes a right-handed coordinate system. In most of the laboratory experiments summarized here, $x$ is along the radial direction $R$

In collisional MHD plasmas, the only non-ideal electric field is due to collisional resistivity, $\eta _{s} \boldsymbol{j}$, while ions and electrons are closely coupled to behave as a single fluid, moving at the MHD fluid velocity $\boldsymbol{V}$. In contrast, collisional resistivity is negligible in collisionless plasmas where a non-ideal-MHD electric field must come from other terms on the RHS of Eq. (1). In such plasmas, ions and electrons decouple from each other as they approach the current sheet. Ions get demagnetized in a larger ion diffusion region (IDR) while electrons get demagnetized closer to the X-line in a smaller electron diffusion region (EDR), see Fig. 1. In general, the second and third terms on the RHS of Eq. (1), $\boldsymbol{j} \times \boldsymbol{B}/{en} - \boldsymbol{\nabla}p_{e}/{en}$, are responsible for non-ideal-MHD electric field in the IDR depending on the guide field strength, while the last two terms are responsible for non-ideal electric field in the EDR. Below we review the laboratory studies of kinetic structures in both the IDR and the EDR, in comparison with space measurements and numerical simulations, with or without a guide field, as well as with and without symmetries between the two upstream reconnection regions.

### IDR Structures Without a Guide Field

When the guide field is negligible, the reconnection electric field $E_{y}$ is perpendicular to the magnetic field, which is mostly within the reconnection plane of $(z,x)$. A natural candidate to balance the required non-ideal-MHD electric field perpendicular to the local magnetic field is the second term on the RHS of Eq. (1), $\boldsymbol{j} \times \boldsymbol{B}/{en}$, which is often called the Hall term. The Hall term originates from the differences in the in-plane ion and electron motions as expected in the IDR. Since such motions preserve symmetry between both upstreams and also both downstreams (unless distant asymmetries are imposed; see below), a quadrupolar structure in the out-of-the-plane (Hall) magnetic field component $B_{y}$ on the scale of the ion skin depth has been predicted theoretically (Sonnerup 1979; Terasawa 1983) and numerically (Birn et al. 2001, and references therein).

In addition to the inductive reconnection electric field in the out-of-the-plane direction, $E_{y}$, there may exist an in-plane electric field $\boldsymbol{E}_{\text{in-plane}}$. At the outer scales (regions outside of the IDR) where ideal MHD applies, the RHS of Eq. (1) vanishes, resulting in $\boldsymbol{E}_{\text{in-plane}} = - (\boldsymbol{V} \times \boldsymbol {B})_{\text{in-plane}}$. Without a guide field, $E_{\text{in-plane}} = - V_{y}B$, which vanishes unless there exists a significant out-of-the-plane flow, $V_{y}$.

However, a significant $\boldsymbol{E}_{\text{in-plane}}$, called the Hall electric field, arises even without an ion flow $V_{y}$ in the IDR. This is because in the IDR, but outside of the EDR, only ion dynamics are dissipative (see Sect. 3.2 on the effects on energy dissipation) and electron dynamics are ideal. Therefore, $\boldsymbol{E} \approx -\boldsymbol{V_{e}} \times \boldsymbol {B}$ and $E_{\text{in-plane}} \approx - V_{ey}B \approx j_{y}B/en$. It also follows that $\boldsymbol{E}\cdot \boldsymbol{B} \approx 0$, and without a guide field, $\boldsymbol{E}_{\text{in-plane}}\cdot \boldsymbol{B} \approx 0$. In other words, $\boldsymbol{E}_{\text{in-plane}}$ is perpendicular to the local magnetic field everywhere, which by symmetry must have a quadrupolar structure around X-line, consistent with numerical predictions (e.g. Shay et al. 1998). By the virtue of Faraday’s Law in quasi-steady state ($\partial B_{y}/ \partial t \approx 0$), $\boldsymbol{E}_{\text{in-plane}}$ is curl-free and can be well represented by an electrostatic potential, $\boldsymbol{E}_{\text{in-plane}} \approx -\boldsymbol{\nabla} \phi $. Therefore $\phi $ must have a saddle-type quadrupolar structure determined by the significant out-of-the-plane $j_{y}$ in the IDR.

The presence of both $\phi $ and $B_{y}$ in the IDR enables fast reconnection by diverting a significant amount of incoming magnetic energy directly downstream in the outflow direction via the Poynting vector $E_{x}B_{y}/\mu _{0}$, without having to pass through the X-line. Note here that the electric field normal to the current sheet $E_{x}$ is part of $\boldsymbol{E}_{\text{in-plane}}$ and peaks along the separatrix with a width on electron scales in the EDR and extending to the ion scales further downstream (Chen et al. 2008). Over time, the depleted total pressure at the X-line pulls in more upstream magnetic pressure leading to the open-outflow geometry necessary for fast reconnection (Liu et al. 2022). This magnetic structure of the open-outflow geometry is consistent with the earlier physics explanation of fast reconnection based on whistler dynamics which involves only electrons in the IDR (e.g. Shay and Drake 1998). The prediction of both Hall magnetic and electric fields motivated an intensive search for such field structures as first evidence of fast collisionless reconnection.

#### Symmetric Anti-Parallel Reconnection

A textbook example measurement of the Hall magnetic and electric structures was by the Polar spacecraft (Mozer et al. 2002), where a bipolar signature for both $B_{y}$ and $E_{x}$ was detected as the spacecraft traversed across a current sheet in one of the outflows of a rare event of symmetric, anti-parallel reconnection in Earth’s magnetopause. Later, with the multiple spacecraft of Cluster, 2D structures of Hall magnetic and electric fields were mapped statistically around the X-line in Earth’s magnetotail (Eastwood et al. 2010).

**Fig. 2:**
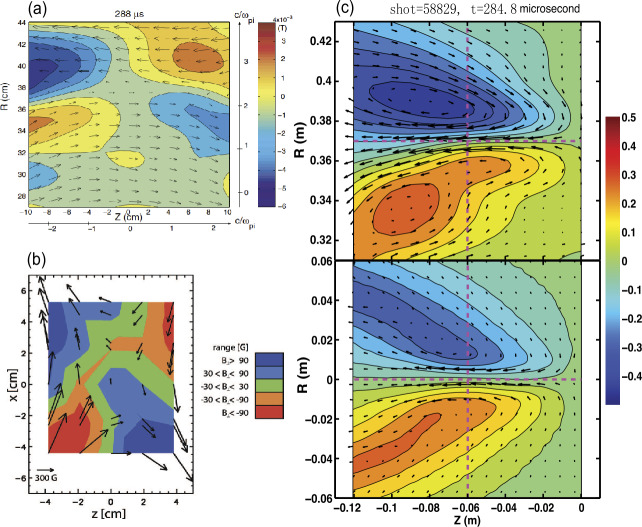
Measured instantaneous quadrupolar structure of the out-of-the-plane magnetic field component during anti-parallel collisionless reconnection. (a) data from Magnetic Reconnection eXperiment or MRX (Ren et al. 2005); (b) data from Swarthmore Spheromak eXperiment or SSX (Brown et al. 2006); (c) comparison between MRX data (top panel) and 2D PIC simulation using corresponding parameters (bottom panel) in one half of the reconnection plane showing excellent agreements on ion scales (Ji et al. 2008). Arrows indicate electron flow velocity

**Fig. 3:**
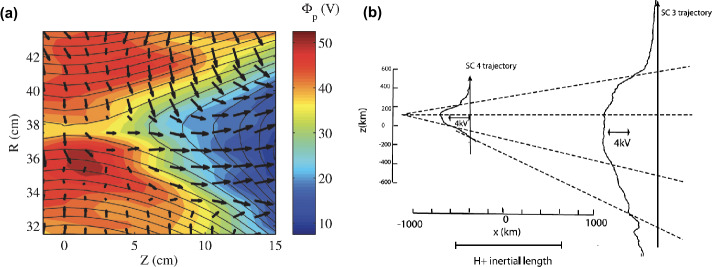
(a) Measured 2D Hall electric potential and ion in-plane flow in MRX during anti-parallel reconnection where half of the saddle-type quadrupolar structure is shown. Adapted from Yamada et al. (2015). (b) Measured Hall electric potential by two Cluster spacecraft during a magnetotail reconnection event, consistent with the expectation that the potential is deeper and wider further from the X-line. Here $X$ is along the reconnecting field direction while $Z$ is the direction across current sheet. Adapted from Wygant et al. (2005)

Aiming to go beyond the 1D measurements by spacecraft, an effort was made in laboratory experiments to directly capture instantaneous 2D quadrupolar structures in $B_{y}$ during anti-parallel reconnection. Figure 2(a) and (b) show the first such measurements from Magnetic Reconnection eXperiment or MRX (Ren et al. 2005) and Swarthmore Spheromak eXperiment or SSX (Brown et al. 2006), respectively. Furthermore, quantitative comparisons were made between MRX and 2D PIC simulations using corresponding parameters, showing excellent agreements on ion scales (Ji et al. 2008), see Fig. 2(c). Since ions control the overall reconnection rate in collisionless reconnection (Biskamp et al. 1995; Hesse et al. 1999), the convergence on the ion-scale kinetic structures between numerical prediction, laboratory experiment and space measurement essentially validated the concept of collisionless fast reconnection. In addition, since collisionality can be actively controlled in the laboratory, continuous transition has been demonstrated from slow Sweet-Parker collisional reconnection (Ji et al. 1998) without a significant $B_{y}$ structure to fast collisionless reconnection with a significant $B_{y}$ structure (Yamada et al. 2006). The Hall electric potential $\phi $ was also simultaneously measured by multiple spacecraft in the magnetotail on the ion scale at downstream (Wygant et al. 2005), and on the electron scale across the current sheet (Chen et al. 2008). The structure is consistent with the 2D measurements in MRX where half of the saddle-type quadrupolar potential structure is shown, see Fig. 3.

#### Asymmetric Anti-Parallel Reconnection

Magnetic reconnection in nature often occurs with significant differences in the density, temperature, and magnetic field strength across the current sheet. A best example of this asymmetric reconnection is reconnection at the magnetopause (Mozer and Pritchett 2011), where the density ratio across the current sheet ranges from 10–100 and a magnetic field strength ratio of 2–4. The asymmetry is expected to significantly alter the structure of the diffusion regions as well as scaling of the reconnection process (e.g. Cassak and Shay 2007).

**Fig. 4:**
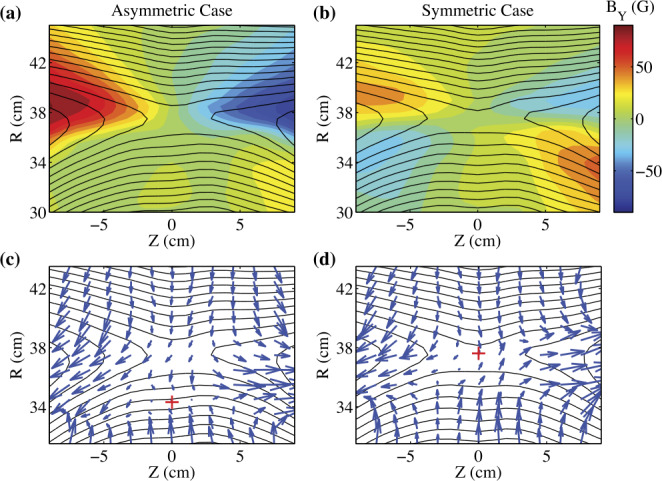
2-D profiles of the out-of-plane magnetic field ($B_{y}$) with contours of the poloidal flux for asymmetric (a) and symmetric (b) cases. Compared to the symmetric case, the quadrupole magnetic field component is enhanced on the high-density side ($R>37.5\text{ cm}$) and suppressed on the low-density side ($R<37.5\text{ cm}$). Black lines indicate contours of the poloidal magnetic flux, which represent magnetic field lines. In-plane ion flow vector profiles for asymmetric (c) and symmetric (d) cases. For the asymmetric case, the ion inflow stagnation point is shifted to the low-density side. The upstream density ratio ($n_{1}/n_{2}$) for the asymmetric case is about 6, while it is about 1.2 for the symmetric case. Figure from Yoo et al. (2014b)

**Fig. 5:**
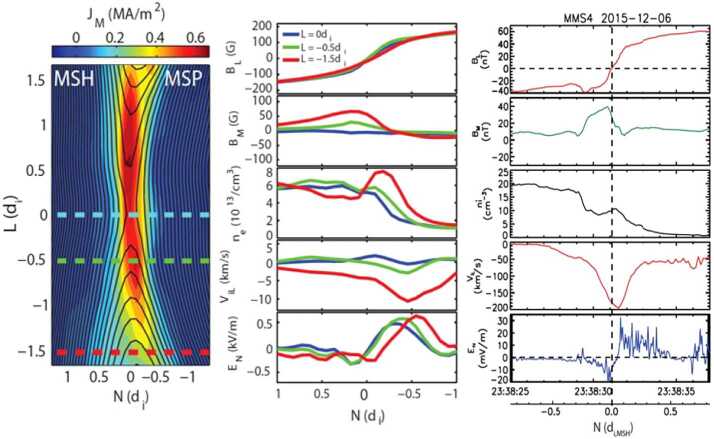
Comparisons of various profiles across asymmetric reconnection current sheets between MRX and MMS. Here the $LMN$ coordinates correspond to the $ZYX$ coordinates. (left panel) 2-D profiles of reconnecting field lines and out-of-the-plane current density in MRX. (middle panel) Cross-current-sheet profiles of magnetic field, density, ion outflow and in-plane electric field at three different locations marked in the left panel. (right panel) Cross-current-sheet profiles of the same quantities during a magnetopause asymmetric reconnection event observed by MMS on December 6, 2015

In the laboratory, reconnection with a strong density asymmetry across the current sheet has been extensively studied and compared to space observations at the subsolar magnetopause (Yoo et al. 2014b, 2017; Yamada et al. 2018). The ratio of the two upstream densities ranges from 5 to 10. It has been shown that strong density asymmetry alters the electric and magnetic field structures in the diffusion regions. In the IDR, the uniform reconnection electric field $E_{y}$ is approximately balanced by the Hall term $\boldsymbol{j}_{\text{in-plane}} \times \boldsymbol{B}/en$ on both upstreams. The asymmetry in density has to be compensated by an asymmetry in $\boldsymbol{j}_{\text{in-plane}}$ since the in-plane magnetic field components are similar, while the pressure balance is maintained by temperature asymmetry. The much larger $\boldsymbol{j}_{\text{in-plane}}$ significantly enlarges $B_{y}$ on the higher density side so that the quadrupolar structure becomes almost bipolar, as shown in Fig. 4(a) and (b) (Yoo et al. 2014b). In contrast, the in-plane electric field is much larger on the low density side since $\boldsymbol{E}_{\text{in-plane}} \approx j_{y}B/en$ where $j_{y}$ and $B$ are similar between the two upstreams. As a result, the in-plane bipolar electrostatic field becomes almost unipolar (Yoo et al. 2017). All these features agree with space observations (e.g. Mozer and Pritchett 2011; Burch et al. 2016). Figure 5 shows excellent agreements between MRX and example MMS measurements at Earth’s magnetopause on profiles of magnetic field components, density, ion outflow, and in-plane electric field.

Strong density asymmetry also causes a shift of the electron and ion inflow stagnation points (Yoo et al. 2014b; Yamada et al. 2018). The ion inflow stagnation point is the location where the in-plane ion flow velocity vanishes. As shown in Fig. 4(c) and (d), the ion inflow stagnation point is shifted to the low-density side by about 3 cm (∼0.5 $d_{i}$; $d_{i}$ is the ion skin depth) for the asymmetric case, while it is very close to the X-point for the symmetric case.

**Fig. 6:**
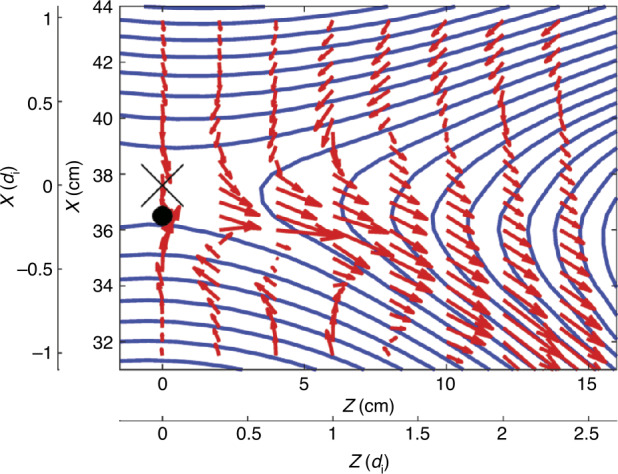
Electron dynamics observed during asymmetric reconnection in MRX. In the reconnection plane, electrons flow together with reconnecting field lines. The X marker at $(R, Z) = (37.6, 0)$ is the X-line and the black circle denotes the stagnation point of in-plane electron flow. Figure from Yamada et al. (2018)

The electron inflow stagnation point is also shifted to the low-density side, as shown in the Fig. 6. The stagnation point denoted by the black dot is shifted by about 1 cm, which is about 0.15 $d_{i}$. These shifts are caused by the imbalance in the electron and ion inflows due to the density asymmetry. This overshooting of electrons from the magnetosheath (high-density) side is consistent with the well-known crescent-shape electron distribution function near the stagnation point (Hesse et al. 2014), which is observed by MMS (Burch et al. 2016).

**Fig. 7:**
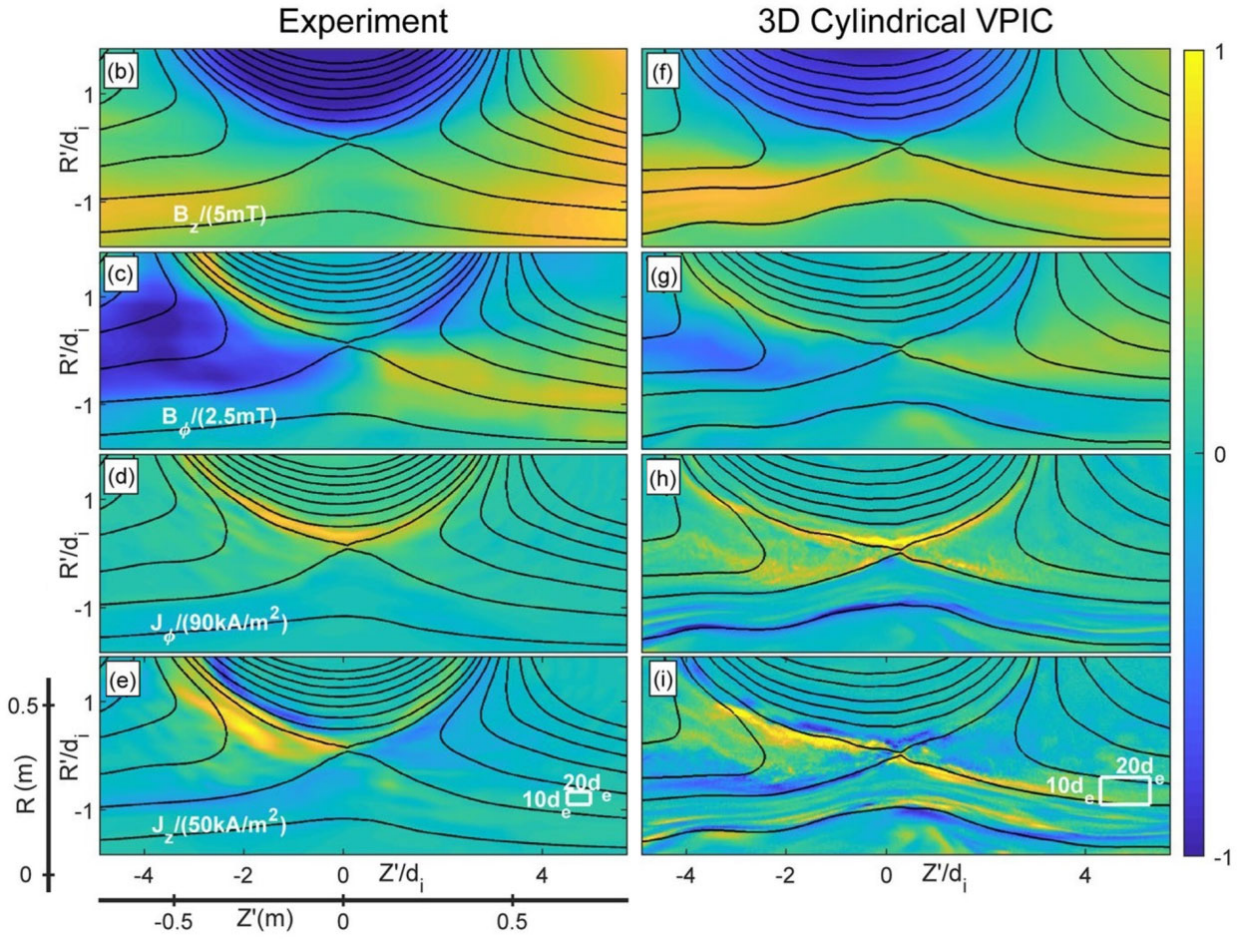
(Panel b-e) Magnetic field and current components recorded in TREX during reconnection. (Panel f-i) Matched 3D kinetic simulation results reproducing the experimental results. After Greess et al. (2021)

The TREX experiment also explored asymmetric anti-parallel reconnection with the plasma density at large radii inflow being suppressed by a factor of about 4. Numerically, the TREX configuration was implemented in the cylindrical version of the VPIC code (Bowers et al. 2009), where properly scaled current sources increasing over time were added at the drive coil locations. Initial density and magnetic field profiles were set at the simulation based on experimental data. As shown in Fig. 7, magnetic field and current structures similar to those of MRX are observed, and reproduced with remarkable agreement through matching numerical simulations (Olson et al. 2021; Greess et al. 2021).

### IDR Structures with a Guide Field

Anti-parallel reconnection is a rather special magnetic geometry in nature, whereas reconnection occurs often with a finite guide field $B_{g}$. With the addition of $B_{g}$, the reconnecting field lines meet at an angle less than $180^{\circ}$, and a sufficiently strong guide field modifies the reconnection process by magnetizing the electrons and ions in the layer. The characteristic kinetic scale across the collisionless current sheet transitions from ion skin depth to ion sound Larmor radius ($\rho _{s}$) as $B_{g}$ increases.

A finite $B_{g}$ also introduces an in-plane electric field structure at the outer ideal scales even without a significant $V_{y}$. This is because in this case $E_{\text{in-plane}}=V_{\text{in-plane}} B_{g}$ where $V_{\text{in-plane}}$ is the in-plane flow due to reconnection. This $E_{\text{in-plane}}$ is required to satisfy the ideal MHD condition $\boldsymbol{E} \cdot \boldsymbol{B}=E_{y}B_{g} + \boldsymbol{E}_{\text{in-plane}} \cdot \boldsymbol{B}=0$ as the reconnection electric field $E_{y}$ now has a parallel component which can extend over a large area. At upstream where the *reconnecting* component $B_{z}$ dominates over the *reconnected* component $B_{x}$, $E_{z} \approx -E_{y} (B_{y}/B_{z}) $ can even dominate the reconnection electric field $E_{y}$ under strong-guide field conditions. Correspondingly, in the downstream where $B_{z}$ is small, $E_{x} \approx -E_{y} (B_{y}/B_{x})$. As before, under quasi-steady conditions ($\partial B_{y}/\partial t \approx 0$) the in-plane electric field is well represented by a quadrupolar potential structure, $\boldsymbol{E}_{\text{in-plane}} = -\nabla \phi $. This potential structure, in turn, drives $\boldsymbol{E} \times \boldsymbol{B}$ drift for both electrons and ions to support the required in-plane, incompressible reconnection flow $\boldsymbol{V}_{\text{in-plane}}$. This quadrupolar potential structure on the outer scales was observed in the VTF (Egedal and Fasoli 2001; Egedal et al. 2003) with a strong guide field and shown to balance the global reconnection electric field in the upstream, as well as interact with global MHD modes that drive reconnection (Katz et al. 2010). However, this quadrupolar potential structure on the outer ideal scales has not been reported by space measurements.

**Fig. 8:**
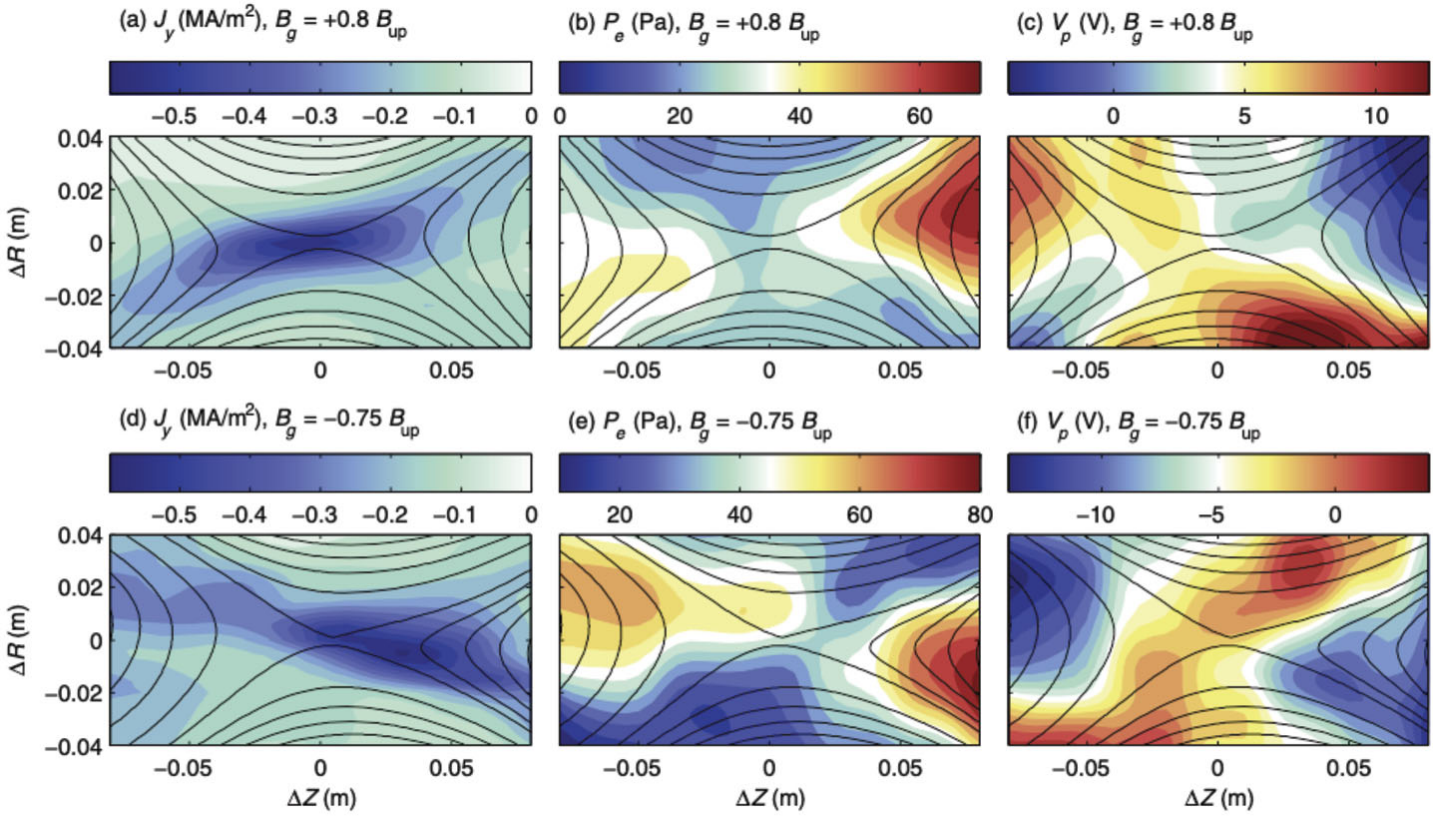
2-D profile data showing observations of quadrupolar pressure variation during guide field magnetic reconnection. (a,d) Plasma current profile; (b,e) Plasma pressure; (c,f) Plasma potential. Between (a-c) and (d-f) the sign of the guide field was reversed, leading to a change in the orientation of the quadrupolar profiles. After Fox et al. (2017)

This quadrupolar potential structure persists from the outer ideal scales to the IDR with a characteristic scale of $\rho _{s}$ during guide field reconnection. When approaching $\rho _{s}$ scale, in addition to the *incompressible* $\boldsymbol{u}_{E}=\boldsymbol{E}\times \boldsymbol{B}/B^{2}$ drift, the in-plane ion polarization drift, $\boldsymbol{u}_{p}=(m_{i}/eB^{2})(\boldsymbol{u}_{E}\cdot \boldsymbol{\nabla})\boldsymbol{E}_{ \text{in-plane}}$, becomes increasingly important. Here $m_{i}$ is ion mass. This cross-field ion polarization drift is *compressible*, and it can generate density variation with electrons moving along the field line to satisfy quasineutrality (Kleva et al. 1995). Combined with the continuity equation, $(\boldsymbol{u}_{E} \cdot \boldsymbol{\nabla})n+n \boldsymbol{\nabla} \cdot \boldsymbol{u}_{p}=0$, the predicted density variation obeys $\ln{(n/n_{0})} = (m_{i}/eB^{2})\nabla ^{2}\phi $ with a quadrupolar structure. This density structure develops large electron pressure variations along the field lines until the third term on the RHS of Eq. (1) becomes important so that

2 $$ E_{\parallel}=-\frac{\nabla _{\parallel }p_{e}}{en} \approx - \rho _{s}^{2} \nabla _{\|}\nabla ^{2} \phi $$

to reach a steady state. Since we also have $E_{\parallel}=-\nabla _{\|}\phi $, Eq. (2) implies that the spatial scale of $\phi $ variation is on the order of $\rho _{s}$, the characteristic scale of the IDR with a guide field. The quadrupolar density structure has been directly measured on MRX during guide field reconnection as shown in Fig. 8. Such a structure was originally predicted from two-fluid extended MHD simulations (Aydemir 1992; Kleva et al. 1995). Øieroset et al. (2016) have measured a plasma density variation consistent with such a quadrupolar structure during a current sheet crossing by MMS. The correspondence was observed in a symmetric guide-field reconnection event, and inferred through comparison with simulations. The crossing of the current sheet was sufficiently downstream that only a bipolar variation (half a quadrupole) was observed.

### EDR Structures

The last two terms in Eq. (1) are responsible in collisionless plasmas for magnetic field dissipation within the electron diffusion region or EDR, where electrons are demagnetized typically on the order of electron skin depth ($d_{e}$) or gyro radius ($\rho _{e}$). The EDR is the location where magnetic field lines are finally reconnected from upstream to downstream. In particular, the importance of off-diagonal terms in the electron pressure tensor in the EDR has been predicted theoretically (Vasyliunas 1975; Lyons and Pridmore-Brown 1990), demonstrated numerically (Cai and Lee 1997; Hesse et al. 1999; Pritchett 2001), and explained physically (Kulsrud et al. 2005). Unmagnetized electrons with an in-plane thermal speed $v_{x}$ or $v_{z}$ are subject to free acceleration by the reconnection electric field $E_{y}$, generating a large off-diagonal pressure $P_{xy}$ or $P_{zy}$, respectively, during their transit time in EDR. This manifests as spatial derivatives in the $y$ component of $\boldsymbol{\nabla}\cdot \boldsymbol{\Pi}_{e}$ in Eq. (1). The competing alternative to this dissipation mechanism is the so-called anomalous resistivity based on 3D kinetic instabilities (Papadopoulos 1977, and references therein), which has been used numerically to reproduce the Petschek solution of fast reconnection (Ugai and Tsuda 1977; Sato and Hayashi 1979) since the early phase of reconnection research. There has been evidence from the MMS measurements for the laminar off-diagonal pressure tensor effect (Torbert et al. 2018; Egedal et al. 2018, 2019) and also for the possible importance of anomalous resistivity or 3D effects (Torbert et al. 2016; Ergun et al. 2017; Cozzani et al. 2021).

**Fig. 9:**
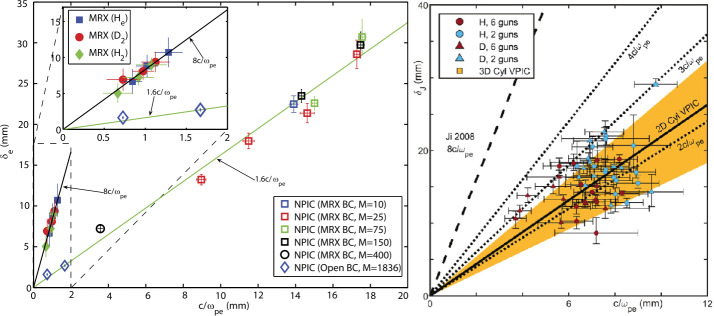
(a) Measured half width of the EDR on MRX compared with 2D PIC simulations in Cartesian geometry (Ji et al. 2008) (b) measured half width of the EDR on TREX compared with 2D (solid line) and 3D (orange region) PIC simulations in cylinderical geometry (Greess et al. 2021)

The EDR has been also identified in anti-parallel reconnection on MRX (Ren et al. 2008) as outgoing electron jets between two quadrants in the $B_{y}$ structure shown in Fig. 2(c). The importance of the off-diagonal pressure tensor in the EDR is closely related to the magnitude and width of such electron jets (Hesse et al. 1999). Compared with 2D PIC simulations in Cartesian geometry, however, the electron jet speed is much slower and the layer half width is 3-5 times thicker (Ji et al. 2008), as shown in Fig. 9(a). This discrepancy persisted even after incorporating finite collisions (Roytershteyn et al. 2010) and 3D effects via Lower Hybrid Drift Waves (LHDW, see later) (Roytershteyn et al. 2013) in the simulations when averaged over the $y$ direction. In contrast, the EDR has been recently studied on TREX and their measured half width agrees well with the predictions by 2D PIC simulations in cylindrical geometry (Greess et al. 2021), shown in Fig. 9(b). 3D effects via LHDW can distort the EDR in the out-of-the-plane direction, weakly broadening the numerical directions of the EDR width [orange region in Fig. 9(b)], but the off-diagonal pressure tensor effect remains dominant at each location.

In addition to the differences in simulation geometries, there are several possibilities to resolve these different results. First, the anti-parallel reconnection in this comparison was driven symmetrically on MRX (Fig. 2) but asymmetrically on TREX (Fig. 7). It is unclear whether symmetry plays a role in determining EDR thickness. Second, the colder ion temperature $T_{i} \ll T_{e}$ at TREX may favor triggering LHDW which can distort the EDR (Roytershteyn et al. 2012), compared with MRX where $T_{i} \sim T_{e}$. Third, there are also differences in measuring the EDR: the “jogging” method in which the EDR is rapidly swept over a 1D probe array in TREX may have higher effective spatial resolutions, but requires that the structures remain in the same shape as confirmed experimentally (Olson et al. 2021), while such a requirement is not needed but the spatial resolution is less effective for the 2D probe array on MRX.

Furthermore, if there is sufficient scale separation between the electron skin depth ($d_{e}$) and Debye length ($\lambda _{D}$) during anti-parallel reconnection, $d_{e}/\lambda _{D}=c/v_{\text{th,e}}>30$, the counter-streaming electron beams in the unmagnetized EDR are unstable to streaming instabilities (Jara-Almonte et al. 2014), possibly leading to efficient dissipation broadening the EDR. Interestingly, this condition is equivalent to $T_{e} < 570\text{ eV}$ which is generally satisfied in space, solar and laboratory plasmas, except in Earth’s magnetotail and also in the typical PIC simulations where laminar anti-parallel reconnection is dominated by electron pressure tensor effects (e.g. Torbert et al. 2018; Egedal et al. 2019). For guide field reconnection, this condition should be revised to $\rho _{e}/\lambda _{D} = \omega _{pe}/\omega _{ce}= (\sqrt{\beta _{e}/2}) d_{e}/\lambda _{D}>30$ implying the importance of electron beta $\beta _{e}$. Obviously, further research is needed to resolve these differences in order to understand better when and how 2D laminar or 3D anomalous effects dominate the dissipation in the EDR.

## Energy Conversion and Partitioning

### Magnetic Energy Dissipation at the X-Point

The primary consequence of magnetic reconnection is the impulsive dissipation of excessive free energy in the magnetic field to plasma charged particles. The energy dissipation near the X-point (inside the EDR) is dominated by electron dynamics, as the electron current is much stronger than the ion current in the EDR. The rate of the energy conversion from magnetic to plasma kinetic energy per unit volume can be quantified by $\boldsymbol{j}\cdot \boldsymbol{E}$. In the EDR this is not much different from the often-used dissipation measure at the electron rest frame $\boldsymbol{j}\cdot \boldsymbol{E}^{\prime}$, where $\boldsymbol{E}^{\prime }= \boldsymbol{E}+\boldsymbol{V}_{e} \times \boldsymbol{B}$ (Zenitani et al. 2011), especially near the X-point where electrons are unmagnetized without significant flow. Thus, we will only discuss the quantity of $\boldsymbol{j}\cdot \boldsymbol{E}$ here for simplicity.

**Fig. 10:**
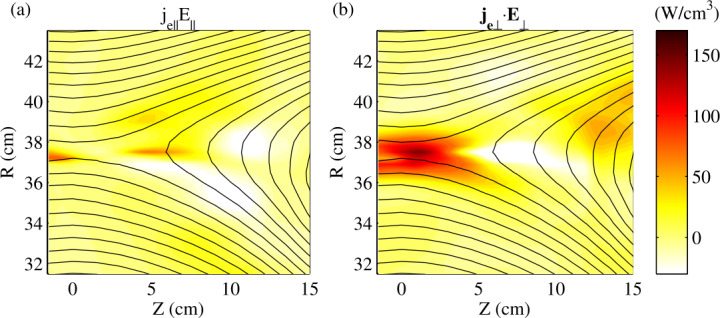
Comparison of two compositions of energy deposition rate measured in MRX for symmetric, anti-parallel magnetic reconnection; (a) $j_{e\parallel}E_{\parallel}$ and (b) $\boldsymbol{j}_{e\perp}\cdot \boldsymbol{E}_{\perp}$. Figure from Yamada et al. (2016)

During anti-parallel reconnection, magnetic energy dissipation near the X-point is dominated by the perpendicular component of $\boldsymbol{j}_{e}\cdot \boldsymbol{E}$, $\boldsymbol{j}_{e\perp}\cdot \boldsymbol{E}_{\perp}$, in both symmetric (Yamada et al. 2014, 2016) and asymmetric cases (Yoo et al. 2017; Yamada et al. 2018). Figure 10 shows a clear dominance of $\boldsymbol{j}_{e\perp}\cdot \boldsymbol{E}_{\perp}$ (panel b) over $j_{e\parallel}E_{\parallel}$ (panel a) near the X-point at $(R,Z) = (37.5, 0)\text{ cm}$ during symmetric, anti-parallel reconnection in MRX. This agrees well with space, where $\boldsymbol{j}_{e\perp}\cdot \boldsymbol{E}_{\perp}$ is strongest near the stagnation point (Burch et al. 2016; Yamada et al. 2018). Furthermore, the perpendicular electric field near the X-point is dominated by the out-of-the-plane reconnection electric field, which can directly accelerate electrons (Zenitani and Hoshino 2001) as shown during a magnetotail reconnection event measured by MMS (Torbert et al. 2018), and also recently during anti-parallel reconnection driven by lasers (Chien et al. 2023) where an accelerated electron beam was detected.

**Fig. 11:**
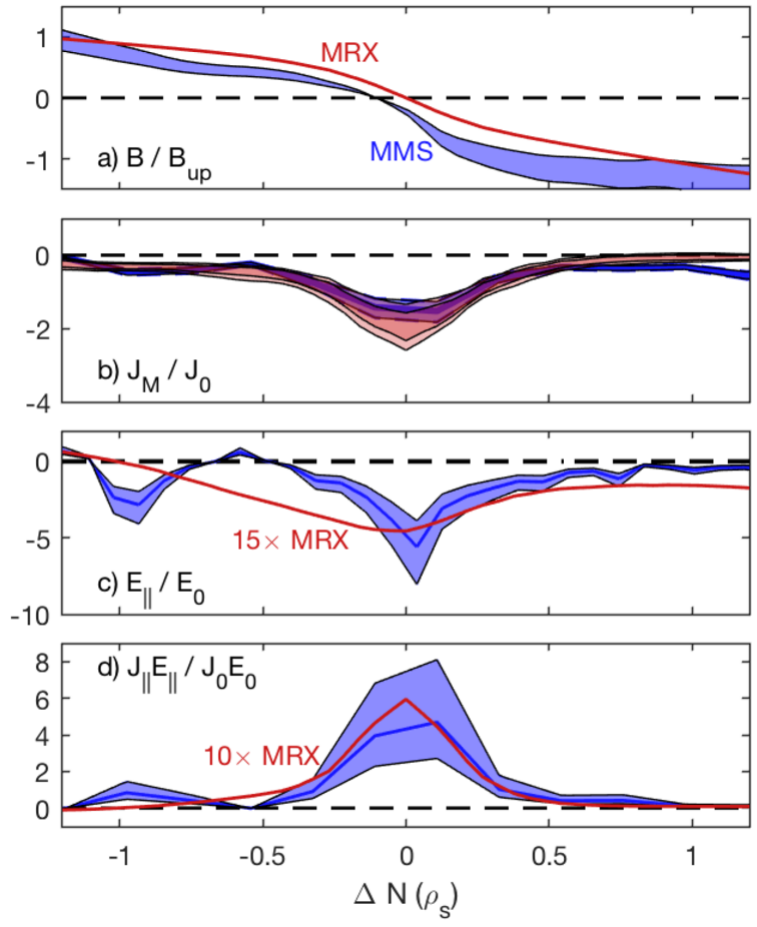
Scaled comparison of MRX (red curves and bands) and MMS (blue bands) data from the event of Eriksson et al. (2016), for cuts across the current sheet of (a) the reconnecting magnetic field, (b) out-of-the plane current density, (c) parallel electric field, and (d) the parallel component of energy dissipation rate. From Fox et al. (2018)

If there is a significant guide field, however, the energy conversion is dominated by the parallel component, $j_{e\parallel}E_{\parallel}$ (Fox et al. 2018; Pucci et al. 2018; Bose et al. 2023), consistent with MMS observation (Wilder et al. 2018). This difference is mainly related to the fact that the energy conversion inside the EDR is mostly through the out-of-plane reconnection electric field. Without a guide field, the reconnection electric field is mostly perpendicular to the magnetic field, while it becomes mostly parallel to the magnetic field with a sizable guide field. Figure 11 shows direct and scaled comparisons between MRX data with a guide field of about 0.6 times the reconnecting field (Fox et al. 2017) and MMS data with a guide field of about 3.5 times the reconnecting field (Eriksson et al. 2016). When normalized properly, the profiles of the magnetic field and current density agree with each other within error bars. A similar conclusion was obtained when compared with another MMS event with lower guide field (Wilder et al. 2018). In both cases $\boldsymbol{j} \cdot \boldsymbol{E}$ in the current sheet is dominated by $j_{\parallel }E_{\parallel}$, consistent with numerical predictions (Pucci et al. 2018). The peak values of the parallel electric field, however, are larger by an order of magnitude in MMS than in MRX. This highlights the importance in our further understanding energy conversion by reconnection (Ergun et al. 2016a), including questions on where these intense parallel electric fields come from and what effects they have on plasma heating and acceleration.

### Energy Conversion

Particle heating and acceleration local to the reconnection region have been directly measured in detail in the laboratory (Hsu et al. 2000; Brown et al. 2002; Stark et al. 2005; Ono et al. 2011; Tanabe et al. 2015; Yoo et al. 2013, 2014b). During anti-parallel reconnection in MRX, whether symmetric or asymmetric, incoming ions from upstream are directly accelerated by the in-plane electrostatic electric field $\boldsymbol{E}_{\text{in-plane}}$ in the IDR (Yoo et al. 2013, 2014b) (see Fig. 3(b)) before they are “remagnetized” further downstream, converting flow energy to thermal energy. Although $\boldsymbol{E}_{\text{in-plane}} \approx -(\boldsymbol{V_{e}} \times \boldsymbol {B})_{ \text{in-plane}}$ is non-dissipative for electrons within the IDR (but outside the EDR), it can energize ions via $\boldsymbol{j}_{i}\cdot \boldsymbol{E}_{\text{in-plane}} \approx en \boldsymbol{V}_{i} \cdot ( \boldsymbol{V_{e}} \times \boldsymbol {B})_{\text{in-plane}}$ (Liu et al. 2022). This has been confirmed experimentally and numerically (Yoo et al. 2014a; Yamada et al. 2018).

During strong guide field reconnection in VTF, ion heating was observed and interpreted (Stark et al. 2005) as magnetic moment conservation being broken due to strong motional variation of the in-plane electric field (Egedal et al. 2003), $(\boldsymbol{v} \cdot \boldsymbol{\nabla}) \boldsymbol{E}_{\text{in-plane}}$. A key dimensionless parameter $e \nabla ^{2} \phi / m_{i} B^{2} \gtrsim 1$ was identified to demagnetize and energize ions (Stark et al. 2005). Ions are heated downstream of magnetic reconnection during plasma merging with a significant guide field (Ono et al. 2011).

Electron heating is mostly localized to the EDR near the X-line during symmetric anti-parallel reconnection as implied by the large value of $\boldsymbol{j}\cdot \boldsymbol{E}$ there (Yoo et al. 2014a) or along the low-density side of separatrices during asymmetric anti-parallel reconnection on MRX (Yoo et al. 2017). While parallel electric field is expected to explain a large fraction of the electron temperature increase (Egedal et al. 2013; Yoo et al. 2017), other mechanisms, such as various wave activities (see below), are not excluded (Ji et al. 2004; Zhang et al. 2023). Electron heating is also measured during guide field reconnection in the electron-only region (Shi et al. 2022) and in the electron-ion region on MRX (Bose et al. 2023). Strong electron heating was observed within the current sheet during plasma merging (Tanabe et al. 2015). These results are in general agreement with MMS results on significant electron energization within the EDR (Eastwood et al. 2020).

Direct measurements of particle acceleration local to the reconnection region are generally difficult in the laboratory, despite many acceleration mechanisms having been proposed and studied intensively numerically (Ji et al. 2022). They include direct acceleration by the reconnection electric field (Zenitani and Hoshino 2001), the parallel electric field (Egedal et al. 2013), Fermi acceleration (Drake et al. 2006), and betatron acceleration (Hoshino et al. 2001). Accelerated electrons along the magnetic field were measured by an energy analyzer (Gekelman and Stenzel 1985) during reconnection, although in a different region. On VTF where reconnection is driven dynamically with a strong guide field, the population of energized tail electrons along the field line were seen to increase by several times, doubling an effective temperature from $\sim 20\text{ eV}$ to up to 40 eV (Fox et al. 2010, 2012). Electron jets at the electron Alfvén speed have been directly detected by Thomson scattering diagnostics during guide field electron-only reconnection (Shi et al. 2022). More recently, non-thermal electrons with energies of $\sim 100T_{e}$ due to the reconnection electric field of anti-parallel reconnection at low-$\beta $ driven by lasers were directly detected with an angular dependence consistent with simulation (Chien et al. 2023). The later supports an astrophysical conjecture to accelerate electrons by reconnection to high energies beyond the synchrotron burnoff limit (Cerutti et al. 2013).

### Energy Partitioning

One of the advantages of laboratory experiments over space measurements is that 2D profiles of key plasma and field parameters can be obtained by repeating measurements over a similar set of discharges. These 2D profiles can be used for a quantitative study of energy conversion and partitioning inside the IDR on MRX (Yamada et al. 2014; Yoo et al. 2017; Bose et al. 2023), where the method of the energy inventory analysis has been explained in detail. The incoming magnetic energy, for example, can be obtained by integrating the corresponding Poynting flux ($E_{y}B_{z}/\mu _{0}$) at the boundary surface. The electron (ion) energy gain can be obtained by integrating $\boldsymbol{j}_{e}\cdot \boldsymbol{E}$ ($\boldsymbol{j}_{i}\cdot \boldsymbol{E}$) over the entire volume of the analysis.

Table 2 summarizes the energy partitioning for three cases in the lab, two cases in numerical simulations, and one case from space measurements. In all cases, the ion energy gain exceeds that of electrons. Compared to antiparallel reconnection, the total energy conversion is less effective for the case with a guide field at a strength comparable to the reconnecting field component. In all cases, both electron and ion energy gain is dominated by an increase in the thermal energy; the flow energy increase is negligible especially for electrons. These results are in general agreement with space observations (Eastwood et al. 2013) which is also listed in the table for comparison, though they carry large uncertainties due to limited available data. Nonetheless, the fact that all these numbers roughly agree with each other suggests that energy conversion and partitioning in locations near the X-line during collisionless reconnection are reasonably quantified.

**Table 2:** Summary of the energy inventory studied in the laboratory for three cases and their counterparts based on PIC simulations for two cases (Yamada et al. 2014; Yoo et al. 2017; Yamada et al. 2018; Bose et al. 2023). Typical errors for these numbers are about 10–20%. The guide field was about 0.7 times the reconnecting field for the guide field reconnection case. One study of space data for a symmetric antiparallel case in Earth’s magnetotail (Eastwood et al. 2013) is also listed despite the large uncertainties in determining incoming magnetic energy and size of the volume (Yamada et al. 2015)

Case	Incoming (MW)	Outgoing	Electron	Ion
Symmetric, antiparallel, lab	1 (1.9 ± 0.2)	0.45	0.20	0.35
Symmetric, antiparallel, PIC	1	0.42	0.22	0.34
Symmetric, antiparallel, space	1	0.1-0.3	0.18	0.39
Asymmetric, antiparallel, lab	1 (1.4 ± 0.2)	0.44	0.25	0.31
Asymmetric, antiparallel, PIC	1	0.43	0.25	0.32
Symmetric, guide field, lab	1 (1.5 ± 0.2)	0.65	0.15	0.29

## Plasma Waves

While magnetic reconnection converts magnetic energy to plasma energy, various free energy sources for waves and instabilities are available especially in or near the diffusion regions and separatrices, such as spatial inhomogeneity, relative drift between ions and electrons (or electric current), or kinetic structures in particles’ velocity distribution functions. This section reviews relevant studies of plasma waves generated in the vicinity of diffusion regions of collisionless reconnection in the laboratory in comparison with space measurements.

### Whistler Waves

One of these types of waves is whistler waves, which can be generated by either electron beams or temperature anisotropy as summarized by Khotyaintsev et al. (2019). During asymmetric reconnection, the separatrix region on the low-density (magnetospheric) side is unstable to lower hybrid drift waves (LHDW) (Krall and Liewer 1971, see below) due to the large density gradient across the magnetic field. This instability enhances the electron transport and heating near the separatrix region (Le et al. 2017). In this region, electrons with a high parallel velocity can be quickly transported to the exhaust region along the turbulent field lines due to LHDW, leaving behind a population of electrons with temperature anisotropy due to a tail with higher perpendicular energy. This temperature anisotropy generates whistler waves around $0.5 f_{\text{ce}}$ near the separatrix on the low-density side (Yoo et al. 2018, 2019).

**Fig. 12:**
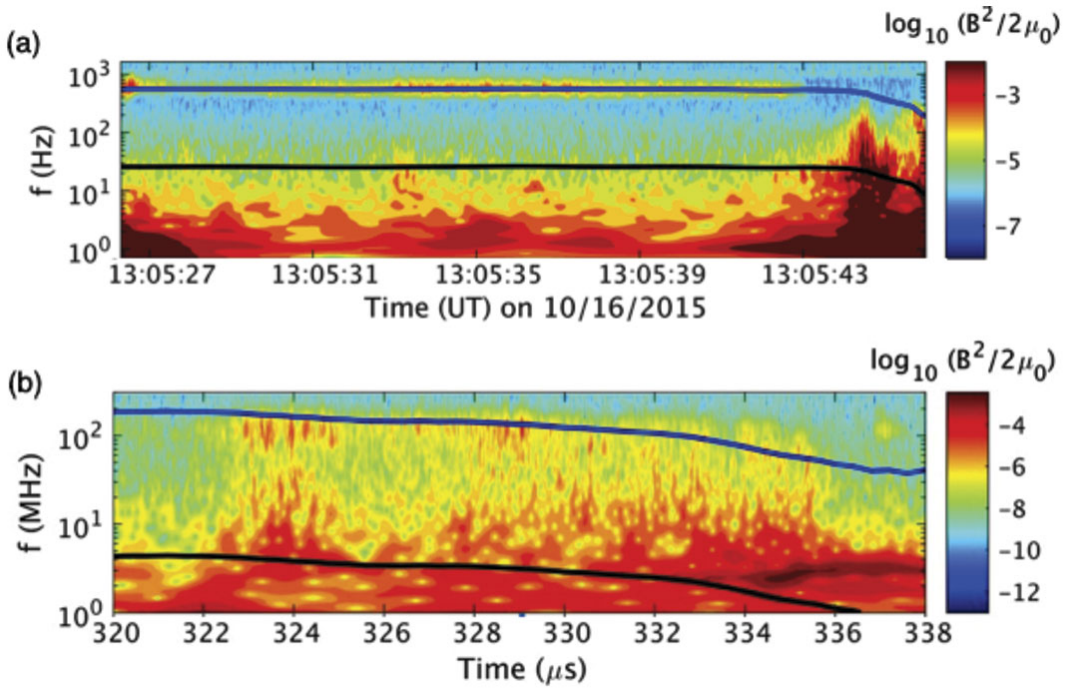
Comparison of the whistler wave activity during asymmetric reconnection observed in space (a) and MRX (b). Blue lines indicate half of the local electron cyclotron frequency ($f_{\text{ce}}$), while black lines indicate the local lower hybrid frequency ($f_{\text{LH}}$). Near the separatrix on the low-density side, whistler waves near 0.5 $f_{\text{ce}}$ are observed. After Yoo et al. (2018)

Figure 12 shows this anisotropy-driven whistler wave observed by MMS (a) and in MRX (b). The color contour shows the energy in fluctuations in the magnetic field. Clear whistler wave activity around the half of the local electron cyclotron frequency ($0.5f_{\text{ce}}$), which is indicated by blue solid lines, is observed in both space and laboratory. In both cases, the measurement location was initially just outside of the separatrix region and moved to the exhaust region around 13:05:43 for the panel (a) and 334 μs for the panel (b). Broad fluctuations mostly below the local lower hybrid frequency ($f_{\text{LH}}$, denoted by black lines) also exist in both measurements. Note that LHDW-driven fluctuations are strongest just before the measurement location enters into the exhaust region. It should be also noted that the whistler wave activity disappears in the exhaust region.

It is worth mentioning that whistler waves were also observed in an earlier reconnection experiment (Gekelman and Stenzel 1984). These waves propagate obliquely with respect to the magnetic field and their amplitudes correlate with the reconnection current. Both of these characteristics are consistent with the observation of electromagnetic LHDW on MRX (Ji et al. 2004), which are explained by a local two-fluid theory (Ji et al. 2005). LHDW will be discussed below in Sect. 4.3.

### Electrostatic Waves

A variety of electrostatic high-frequency waves have also been observed in the laboratory during reconnection events. Above $f_{\text{LH}}$, these waves have multiple names, including R-waves [after the $R=0$ branch in the Clemmow-Mullaly-Allis (CMA) diagram (Stix 1992)], electrostatic whistlers, or Trivelpiece-Gould modes [from early laboratory contexts (Trivelpiece and Gould 1959)]. These waves extend from $\sim f_{LH}$ to $\min (f_{pe}, f_{ce})$. Under most laboratory as well as space conditions, $f_{ce} < f_{pe}$, so the waves exist up to $f_{ce}$. For the waves to be electrostatic $k d_{e} > 1$ must be satisfied, where $k$ is the wavenumber. The electrostatic branch has the dispersion relation $\omega = \omega _{ce} k_{\parallel}/k$, which allows a broadband collection of waves with parallel phase velocities $\omega /k_{\parallel}$ resonant with super-thermal electron populations. At longer wavelength, when $k d_{e} < 1$, these waves transition to the classical electromagnetic whistlers ($\omega = \omega _{ce} d_{e}^{2} k_{ \parallel }k$). At lower frequencies $f \sim f_{\text{LH}}$, the waves increasingly interact with the ions. In those cases, the perpendicular group velocity of the waves becomes very small, so that wave packets can stay localized to regions with energized electrons for efficient growth. Theory predicts that there are multiple sources of free energy which can drive the waves, including beam resonance (inverse Landau damping); gyro-resonance driven by $T_{\parallel }> T_{\perp}$; or gradients in density, temperature, or in fast electron components (Fox et al. 2010). Most interestingly, the waves driven by gradients lead to maximum growth in the lower-hybrid range frequencies ($f \sim f_{LH}$), and are related to quasi-electrostatic lower-hybrid drift waves (see below).

Gekelman and Stenzel (1985) also reported the detection of these waves and suggested that they are generated by the measured energetic electron tail in the 3D velocity space, either by anisotropy mechanisms or inverse Landau damping. High-frequency electrostatic waves were also detected on VTF when guide field reconnection was strongly driven (Fox et al. 2010). This was consistent with a picture where the reconnection events would drive energetic electrons, which in turn would drive waves. The parallel phase speed was observed to be resonant with superthermal electrons, $\omega / k_{\parallel }> v_{te}$. The spectrum typically consisted of a broad spectrum from near $f_{\text{LH}}$ and extending to a very clear cutoff at $f_{ce}$ (Fox et al. 2010).

**Fig. 13:**
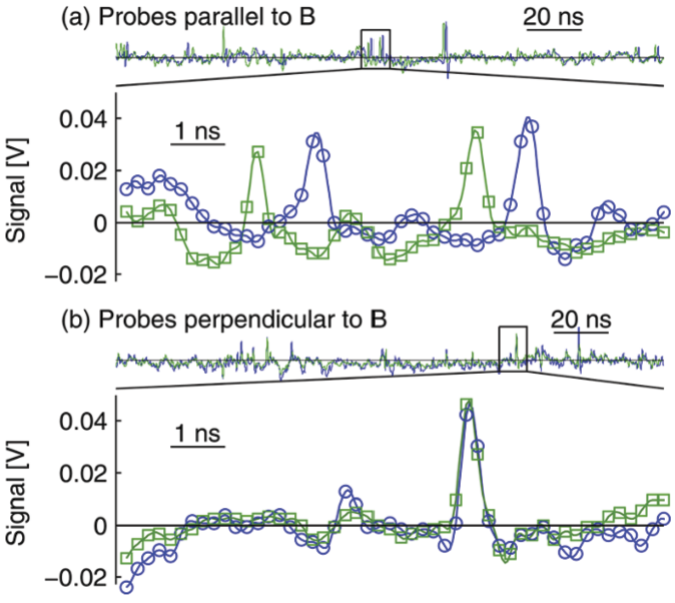
Observation of phase-space-hole electrostatic structures driven during magnetic reconnection events. a) Propagation between two closely-spaced probes parallel to the magnetic field, b) simultaneous observation on two probes oriented perpendicular to the magnetic field. The time delays combined with known probe separate give the typical size and velocity of the electron holes, which is superthermal compared to the electron temperature. From Fox et al. (2012)

Given strong beam components, electrostatic waves can often be driven to very large amplitude, which can lead to the formation of non-linear wave structures. One such mechanism is that the waves can grow to large amplitudes and trap resonant electrons. This leads to so-called “electron phase-space hole” structures, also called Bernstein-Greene-Kruskal (BGK) solitary structures (Bernstein et al. 1957), or electrostatic solitary waves (ESW). The latter have been observed in many places in space including during reconnection events in the magnetopause (Matsumoto et al. 2003) and magnetotail (Cattell et al. 2005), as was summarized recently by Khotyaintsev et al. (2019). These electron phase space holes were directly observed on VTF (Fox et al. 2008, 2012) and indicate that the strong electric fields in the reconnection region pull-out strong beam components of the electron population, exciting these hole structures. Electron holes have also been directly generated in electron-beam experiments (Lefebvre et al. 2010). Figure 13 shows observations of electron hole phenomena during the strong wave turbulence during VTF reconnection events. The structures are positive potential ($\phi > 0$) which is consistent with electron trapping. More recently, ESW or electron space holes have been observed during guide field reconnection within the diffusion region (Khotyaintsev et al. 2020) and in the separatrix (Ahmadi et al. 2022) in the magnetopause where they may play an important role in electron heating.

There is a renewed interest in the ion acoustic wave (IAW) (Papadopoulos 1977, and references therein), which is an unmagnetized short-wavelength electrostatic wave. The IAW can be driven unstable by relative drift between ions and electrons or equivalently electric current, which is expected to be intense around the X-line. Anomalous resistivity based on IAW-like waves has been used to numerically generate Petschek solution fast reconnection since Ugai and Tsuda (1977), Sato and Hayashi (1979). Despite a pioneering laboratory detection during relatively collisional reconnection (Gekelman and Stenzel 1984), however, the importance of IAWs for reconnection has been quickly dismissed due to the widely observed high ion temperature $T_{i} \sim ZT_{e}$, which is known to stabilize IAW via strong ion Landau damping. However, in a very recent laboratory experiment using lasers (Zhang et al. 2023), strong IAW bursts and the associated electron acoustic wave (EAW) bursts were detected by collective Thomson scattering in the exhaust of anti-parallel reconnection where $T_{i} \ll ZT_{e}$ due to high $Z (\sim 18)$ of ions. These IAW and EAW burst were successfully reproduced by PIC simulations showing that strong IAWs generate a double layer, which induces electron two-stream instabilities leading to EAW bursts and electron heating as observed experimentally. These new experimental results are consistent with recent space observations (Uchino et al. 2017; Steinvall et al. 2021) which detected IAWs during reconnection when sufficient cold ions were present, and may be relevant to the outstanding questions on large parallel electric fields measured by MMS (Ergun et al. 2016b). These new results also raised a legitimate question on whether the high ion temperature is a universal observation and thus whether IAW should be dismissed as an anomalous dissipation mechanism in collisionless plasmas. In fact, recent detection of monochromatic IAWs and associated electron heating in solar wind when ions are cold (Mozer et al. 2022) speaks for the needs to revisit this topic, as direct measurements of ion temperature are rare for solar and astrophysical plasmas in general.

### Lower Hybrid Drift Waves and Current Sheet Kinking

Lower hybrid drift waves (LHDWs) have been a candidate for anomalous resistivity and transport in the diffusion region due to their ability to interact with both electrons and ions. The free energy source of LHDWs is the current perpendicular to the magnetic field (Davidson and Gladd 1975). Depending on the local plasma and field parameters, LHDWs may be either quasi-electrostatic (ES-LHDW) (Carter et al. 2001; Hu et al. 2021) or electromagnetic (EM-LHDW) (Ji et al. 2004; Yoo et al. 2014b). With a similar electron temperature and perpendicular current, plasma beta ($\beta $) is the key parameter to determine the type of waves; for low $\beta $ (typically below unity), the ES-LHDW mode propagating nearly perpendicular to the local magnetic field is unstable, while the EM-LHDW mode propagating obliquely to the magnetic field is excited when $\beta $ is high (Yoo et al. 2020).

**Fig. 14:**
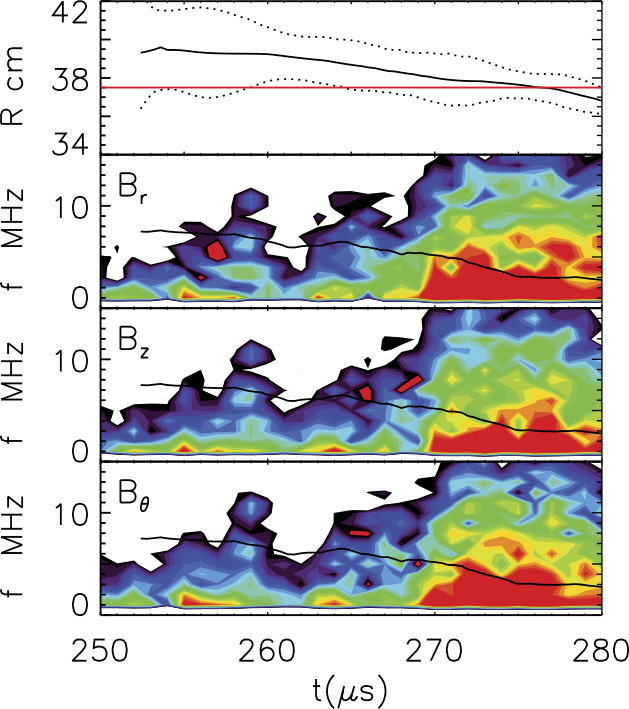
Detection of electromagnetic lower-hybrid drift waves in the current sheet center during anti-parallel reconnection on MRX. Wave powers are color coded (red high and white low) in spectrograms where lower hybrid frequency is indicated by the black line using upstream reconnecting field. Top panel shows the location of the probe (red) and the current sheet (center as black solid line and edges as dashed lines). When the current sheet center moves close to the probe, high-frequency magnetic fluctuations are detected. Figure from Ji et al. (2004)

During anti-parallel reconnection, plasma $\beta $ varies rapidly in the current sheet. At the current sheet edge where $\beta $ is low, the ES-LHDW mode has been observed (Carter et al. 2001; Yoo et al. 2020) consistent with theoretical expectation (Daughton 2003) and space observation by Polar spacecraft (Bale et al. 2002). The obliquely propagating EM-LHDW mode has been observed in the current sheet center where plasma $\beta $ is high and electric current is large (Ji et al. 2004; Yoo et al. 2014b), as well as in the immediate downstream (Ren 2007). An example is shown in Fig. 14 from MRX where large-amplitude electromagnetic waves were detected when the current sheet center moved close to the probe during anti-parallel reconnection (Ji et al. 2004), consistent with numerical simulations (Daughton et al. 2004). Both ES-LHDWs and obliquely propagating EM-LHDWs have also been observed by Cluster spacecraft in a thin current sheet in magnetotail (Zhou et al. 2009) and recently by MMS in magnetopause (Ergun et al. 2017). More recent measurements on MRX show that the EM-LHDW becomes increasingly organized with larger amplitude with guide field (von Stechow et al. 2018). For more measurements of LHDWs in and around diffusion regions in space with varying influence on anomalous resistivity and viscosity, see recent reviews by Khotyaintsev et al. (2019) and Graham et al. (2023).

Many of the observed wave characteristics of EM-LHDWs, such as propagation direction and polarization, have been qualitatively explained by a local two-fluid theory (Ji et al. 2005) as an instability caused by reactive coupling between the backward propagating whistler wave and the forward propagating sound wave when the relative drifts between electrons and ions are large. The wave amplitude has been observed to correlate positively with fast reconnection (Ji et al. 2004), consistent with quasilinear theory on their possible importance for anomalous resisitivity (Kulsrud et al. 2005). The waves have also been reproduced in 3D PIC simulations performed in MRX geometry in a Cartesian coordinate, but they failed to explain the observed broadened width of the EDR (Roytershteyn et al. 2013). Possible solutions to this discrepancy include differences in the simulation geometry and parameters, as well as measurement resolutions as discussed in Sect. 2.3. It is noted that the current sheet kinking that was observed on TREX and associated simulations (Greess et al. 2021) and in space (e.g. Ergun et al. 2019) could result in broadened current sheets due to limited spatial and/or time resolutions.

With a sizable guide field, however, ES-LHDWs can be unstable inside the IDR and EDR, affecting electron and reconnection dynamics. For example, following a multi-spacecraft analysis using Cluster (Norgren et al. 2012), a recent observation (Chen et al. 2020) using MMS showed that strong ES-LHDWs produce non-gyrotropic electron heating and vortical flows inside the EDR of reconnection with a guide field. These electron vortices have been successfully reproduced by corresponding 3D PIC simulations (Ng et al. 2020) and suggest that further reconnection may occur inside the LHDW vortex tubes as dissipation at smaller scales. Other space observations of guide field reconnection show that ES-LHDWs are capable of generating anomalous resistivity between electrons and ions (Yoo et al. 2020; Graham et al. 2022).

**Fig. 15:**
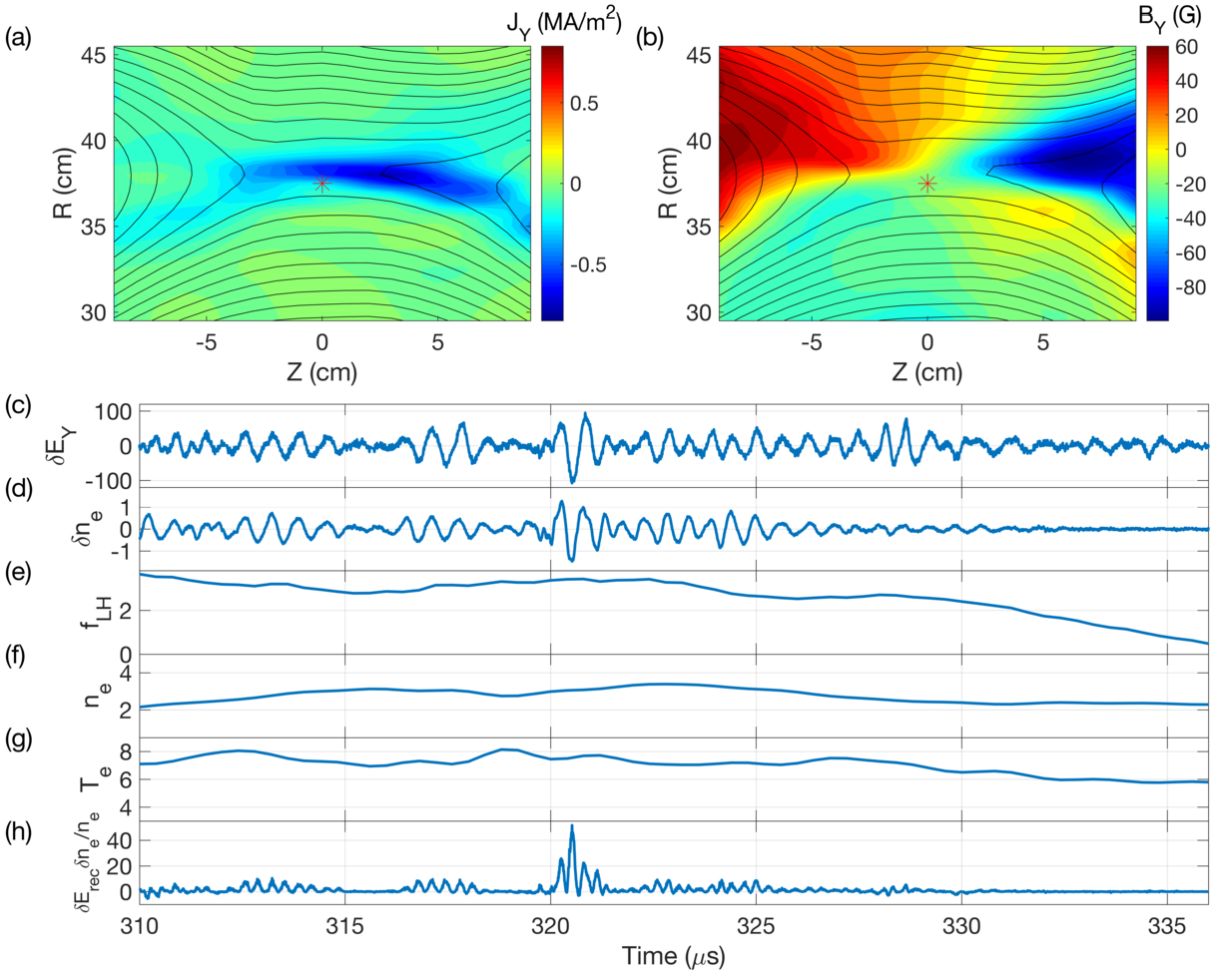
Measured ES-LHDWs. (a,b) Out-of-plane current or magnetic field component (color) with the poloidal flux contours (black lines) representing the magnetic field lines at 326 μs. The red asterisk indicates the location of the probe. The upper side ($R>37.5\text{ cm}$) has a higher density. (c) Time series of $\delta E_{\text{rec}}$ in V/m. Wave activity near the lower hybrid frequency ($f_{\text{LH}}\sim 2\text{ MHz}$) is detected while the probe stays near the reconnection site. The amplitude of the fluctuation is comparable to the mean reconnection electric field ($\langle E_{ \text{rec}} \rangle \sim 100\text{ V}/\text{m}$). (d) Time series of $\delta n_{\text{e}}$ in $10^{13}\text{ cm}^{-3}$ during the quasi-steady reconnection period. Time series of $f_{\text{LH}}$ (e), averaged density ($\langle n_{\text{e}} \rangle $) in $10^{13}\text{ cm}^{-3}$ (f), and electron temperature ($T_{\text{e}}$) in eV (g) are shown. A sharp decrease of $f_{\text{LH}}$ is observed with the approach of the X-point to the probe. Time series of $\delta E_{\text{rec}}\delta n_{\text{e}}/\langle n_{\text{e}} \rangle $ are shown in (h). Positive correlation between $\delta E_{\text{rec}}$ and $\delta n_{\text{e}}$ indicates that the wave is capable of generating anomalous resistivity. Figure from Hu et al. (2021)

Recently, ES-LHDW measurements were revisited on MRX combined with the simultaneous measurements of electron density at the same location (Hu et al. 2021). Figure 15 shows measurements of ES-LHDWs at the edge of the current sheet during anti-parallel reconnection. Panels (a) and (b) show the 2D profile of the out-of-plane current density and magnetic field, respectively. The black lines are contours of the poloidal magnetic flux, representing magnetic field lines. The red asterisk is the location of the probe that measures high-frequency fluctuations in the reconnection electric field (panel c) and electron density (panel d) (Hu et al. 2021). Due to the positive correlation between two fluctuating quantities, the quantity of $\delta E_{y}\delta n_{\text{e}}/\langle n_{\text{e}} \rangle $, which is anomalous resistivity along the out-of-plane direction (Che et al. 2011), becomes positive. These measurements of ES-LHDWs have been further extended on MRX to cases with a sizable guide field demonstrating significant anomalous resistivity and electron heating (Yoo et al. 2023). The initial corresponding 3D simulation show that ES-LHDWs propagating along the outflow are triggered by the difference between electron and ion outflows in regions of low $\beta _{e}$ (Ng et al. 2023), consistent with the MRX experiment results.

## Multiscale Reconnection

The physics of collisionless magnetic reconnection has been studied mostly in locations nearby the local X-line as discussed in the previous sections, such as the IDR and EDR as well as separatrices. If measured in the unit of ion kinetic scales, their distances from the local X-line are not too far. However, the collisionless plasmas in space and astrophysics where reconnection is believed to occur are vastly larger - their normalized sizes have been surveyed (Ji and Daughton 2011) ranging from $\sim 10^{3}$ for Earth’s magnetosphere to $\sim 10^{14}$ for extragalactic jets. In these large plasmas, magnetic reconnection inevitably occurs in the multiple X-line regimes as illustrated in the reconnection phase diagram (Ji and Daughton 2011, 2022).

While there has been abundant evidence for collisionless multiple X-line reconnection in Earth’s magnetopause as Flux Transfer Events (FTEs) (Russell and Elphic 1979) and in the magnetotail as plasmoids (Baker et al. 1984), there have been only relatively few laboratory works in this area with (Stenzel et al. 1986; Ono et al. 2011) or without a guide field (Dorfman et al. 2013; Olson et al. 2016; Jara-Almonte et al. 2016; Hare et al. 2017). When plasmoids form and are subsequently ejected from the current sheet, reconnection tends to proceed in an impulsive and intermittent fashion (Ono et al. 2011; Dorfman et al. 2013; Jara-Almonte et al. 2016), qualitatively consistent with space observations of the non-steadiness of multiscale reconnection (e.g. Chen et al. 2008, 2012; Ergun et al. 2018).

**Fig. 16:**
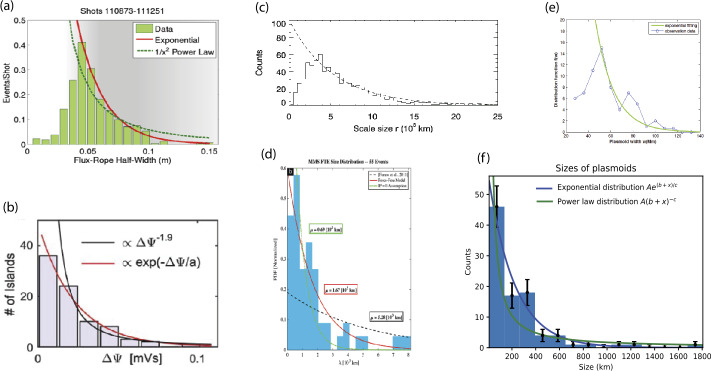
Plasmoid size distributions (a) Dorfman et al. (2014) and (b) Olson et al. (2016) from the lab; (c) Fermo et al. (2011) and (d) Akhavan-Tafti et al. (2018) from the space observation; (e) Guo et al. (2013) from the solar observation (reproduced by permission of the AAS); and (f) Bergstedt et al. (2020) from the space observation. All of them are more consistent with an exponential distribution rather than a power-law distribution

Quantifying non-steady reconnection with multiple X-lines or “turbulent” reconnection is non-trivial. There have been several studies that quantified size distributions of plasmoids, or magnetic structure in general, during multiscale reconnection, as shown in Fig. 16. Two are from the laboratory (Dorfman et al. 2014; Olson et al. 2016), two from Earth’s magnetopause (Fermo et al. 2011; Akhavan-Tafti et al. 2018), one from Earth’s magnetotail (Bergstedt et al. 2020), and one from solar observation (Guo et al. 2013). Other than the last study, the others are on plasmoids on kinetic scales, but all of them are more consistent with an exponential distribution rather than a power-law distribution. It is not surprising to have an exponential distribution on kinetic scales as they are dissipative scales in collisionless plasmas, but it would be a surprise if the exponential distributions also apply to fluid scales, over which the self similar power laws should apply at least in the inertial range. We note that there are interesting statistical *in-situ* studies of heliospheric current sheets (e.g. Eriksson et al. 2022) and flux ropes (Janvier et al. 2014) on a larger scale in the solar wind. The upcoming multiscale experiments, numerical simulations and observatories should shed more light onto these important questions (Ji et al. 2022).

## Future Prospects

A concise review was given on the recent highlights from controlled laboratory studies of collisionless magnetic reconnection on a variety of topics including ion and electron kinetic structures in electromagnetic fields, energy conversion and partitioning, various electromagnetic and electrostatic kinetic plasma waves, as well as plasmoid-mediated multiscale reconnection. While unresolved issues still remain, many of these highlighted results compare well with numerical predictions and space observations, especially by the MMS mission. Thus, it is not an overstatement that the physics foundation of fast reconnection in collisionless plasmas has been largely established, at least within the parameter ranges and spatial scales that were studied.

Nonetheless, there still exist outstanding questions on single X-line collisionless reconnection. The first question is about what dissipates magnetic fields within the EDR when 2D laminar pictures do not apply. We still have cases in the laboratory where the reconnection electric field or the thickness of the EDR is not fully accounted for (Ji et al. 2008; Roytershteyn et al. 2013), while in space we also have cases where 2D laminar reconnection pictures do not tell the whole story (e.g. Cozzani et al. 2021). Does anomalous resistivity exist in its conventional forms, as hinted by electrostatic LHDWs observed during guide field reconnection (Yoo et al. 2023) or by IAWs observed recently during anti-parallel reconnection at low ion temperature (Zhang et al. 2023)? Alternatively, do anomalous effects manifest as kinking of otherwise laminar 2D reconnecting current sheets (Greess et al. 2021) or is anomalous resisitivity cancelled by anomalous viscosity leaving no wave dissipative effects in the EDR (Graham et al. 2022)? Further research using well-controlled experiments with adequate diagnostics, supported by matching numerical simulations, is needed to settle this long standing question.

Another outstanding question is about how magnetic energy is dissipated to a combination of flow, thermal and non-thermal energies of electrons and ions, as a function of field geometry, symmetry, and upstream plasma $\beta $. Substantial progress has been made on this subject with laboratory experiments, numerical simulations, and space observation, as summarized in Table 2 in terms of energy partitioning, but there remain a number of unanswered questions, especially on particle acceleration. Recent progress in directly detecting electrons accelerated by the reconnection electric field (Chien et al. 2023) and non-thermal electrons by Thomson scattering (Shi et al. 2022) is an encouraging sign that more results are coming. The predicted scaling of electron heating and acceleration by the parallel electric field with regard to upstream $\beta $ (Le et al. 2016) is in agreement with certain spacecraft observations (Oka et al. 2023), but its laboratory study sensitively depends on plasma collisionality (Le et al. 2015). High Lundquist number regimes offered by the upgraded TREX (Olson et al. 2016) and the upcoming Facility for Laboratory Reconnection Experiments or FLARE (Ji et al. 2018, 2022) will allow first laboratory accesses to the collisionless regimes required to study this important issue of collisionless reconnection.

Looking further into the future, laboratory access to multiscale regimes of magnetic reconnection is an important step as guided by the reconnection phase diagram (Ji and Daughton 2011, 2022). In addition to high Lundquist numbers, space and astrophysical plasmas have large normalized plasma system sizes, significantly expanding the parameter space over which global fluid scales and local kinetic scales are coupled. The solar corona is an excellent example where the typical mean-free path of thermal particles is much longer than any kinetic scales so that locally physics is collisionless or kinetic, while the mean-free path is much shorter than system sizes so that globally physics is collisional or fluid-like. How does multiscale physics across fluid and kinetic scales operate self-consistently in this regime to generate solar flares as observed, in terms of their impulsive onset and energetic consequences on thermal heating and particle acceleration? Answering multiscale physics questions like this requires going far beyond what has been traditionally done in reconnection research in which the detailed dynamics are studied around local X-lines based on either fluid or kinetic physics.

Statistical properties of multiscale physics need to be quantified in order to identify self-similar behavior across scales. In the case of plasmoid-mediated multiscale reconnection, despite theoretical advances in predicting power-law scaling of plasmoid sizes (e.g. Uzdensky et al. 2010; Huang and Bhattacharjee 2012; Pucci and Velli 2014; Comisso et al. 2016; Majeski et al. 2021), no power-laws have been found from the laboratory or space data thus far. This may be due to the fact that data used are close to dissipative kinetic scales, and thus the accessibility of data on fluid scales is critical. To simultaneously study fluid and kinetic physics, especially under realistic conditions in 3D, effectively exploiting new exascale computing capabilities is crucial (Ji et al. 2022), as highlighted by a recent example in modeling Earth’s magnetotail (Palmroth et al. 2023). In addition, exascale computers will permit fully kinetic simulations to more closely match important dimensionless parameters, such as the ion to electron mass ratio ($m_{i}/m_{e}$) and the ratio of the electron skin depth to the electron Debye length ($d_{e}/ \lambda _{D}$), both of which influence the spectrum and nature of instabilities present with reconnection layers (Jara-Almonte et al. 2014) (see Sect. 2.3). We anticipate that exascale computing will permit larger system sizes ($S$, $L/d_{i}$) and permit 3D global kinetic modeling of laboratory experiments. Furthermore, to process a huge amount of existing and new observational, numerical, and laboratory data for statistical studies, there exist promising opportunities to use novel techniques based on data science such as machine learning (e.g. Bergstedt and Ji 2023).

One of the direct consequences of multiscale collisionless reconnection is its ability to accelerate particles into power-law distributions which are often observed during reconnection events. There has been a recent surge of theoretical and numerical work on this subject including reconnection under extreme conditions in astrophysics using kinetic models (e.g. Dahlin 2020; Li et al. 2021; Guo et al. 2020, and references therein) and MHD models (Arnold et al. 2021; Majeski and Ji 2023); however, there have been no laboratory counterparts on this subject. It is imperative to develop new platforms (e.g. Chien et al. 2023) for such studies as well as new diagnostics (e.g. Fox et al. 2010; Shi et al. 2022) to detect accelerated non-thermal particles in laboratory experiments, including upcoming multiscale experiments such as FLARE (Ji et al. 2018, 2022). A concerted effort from exascale modeling, data science, as well as from the scheduled or proposed multiscale space missions such as HelioSwarm (Klein et al. 2023) and Plasma Observatory (Retinò et al. 2022) is critical to address these important questions.
